# Sequencing and Description of the Mitochondrial Genome of *Orthopodomyia fascipes* (Diptera: Culicidae)

**DOI:** 10.3390/genes15070874

**Published:** 2024-07-03

**Authors:** Fábio Silva da Silva, Bruna Laís Sena do Nascimento, Ana Cecília Ribeiro Cruz, Sandro Patroca da Silva, Carine Fortes Aragão, Daniel Damous Dias, Lucas Henrique da Silva e Silva, Lúcia Aline Moura Reis, Hanna Carolina Farias Reis, Liliane Leal das Chagas, José Wilson Rosa Jr., Durval Bertram Rodrigues Vieira, Roberto Carlos Feitosa Brandão, Daniele Barbosa de Almeida Medeiros, Joaquim Pinto Nunes Neto

**Affiliations:** 1 Graduate Program in Parasitary Biology in the Amazon Region, Center of Biological and Health Sciences, State University of Pará, Belém 66095-663, Brazil; fabiodasilva@iec.gov.br (F.S.d.S.); anacecilia@iec.gov.br (A.C.R.C.); damous1994@gmail.com (D.D.D.); biohenriquesilva@hotmail.com (L.H.d.S.e.S.); luciaalinereis@gmail.com (L.A.M.R.); hanna_carolina@hotmail.com (H.C.F.R.); danielemedeiros@iec.gov.br (D.B.d.A.M.); 2 Evandro Chagas Institute—IEC/MS/SVSA, Department of Arbovirology and Hemorragic Fevers, Ananindeua 67030-000, Brazil; brunanascimento@iec.gov.br (B.L.S.d.N.); sandrosilva@iec.gov.br (S.P.d.S.); carinearagao@iec.gov.br (C.F.A.); lilianechagas@iec.gov.br (L.L.d.C.); josejr@iec.gov.br (J.W.R.J.); durvalvieira@iec.gov.br (D.B.R.V.); robertobrandao@iec.gov.br (R.C.F.B.)

**Keywords:** Culicidae, mitogenome, molecular taxonomy, mosquitoes, phylogenetics

## Abstract

The genus *Orthopodomyia* Theobald, 1904 (Diptera: Culicidae) comprises 36 wild mosquito species, with distribution largely restricted to tropical and temperate areas, most of which are not recognized as vectors of epidemiological importance due to the lack of information related to their bionomy and involvement in the cycle transmission of infectious agents. Furthermore, their evolutionary relationships are not completely understood, reflecting the scarcity of genetic information about the genus. Therefore, in this study, we report the first complete description of the mitochondrial genome of a Neotropical species representing the genus, *Orthopodomyia fascipes* Coquillet, 1906, collected in the Brazilian Amazon region. Using High Throughput Sequencing, we obtained a mitochondrial sequence of 15,598 bp, with an average coverage of 418.5×, comprising 37 functional subunits and a final portion rich in A + T, corresponding to the control region. The phylogenetic analysis, using Maximum Likelihood and Bayesian Inference based on the 13 protein-coding genes, corroborated the monophyly of Culicidae and its two subfamilies, supporting the proximity between the tribes Orthopodomyiini and Mansoniini, partially disagreeing with previous studies based on the use of molecular and morphological markers. The information generated in this study contributes to a better understanding of the taxonomy and evolutionary history of the genus and other groups of Culicidae.

## 1. Introduction

Mosquitoes (Diptera: Culicomorpha: Culicoidea: Culicidae) [1,2] comprise a monophyletic taxon consisting of around 3700 formally recognized species and are taxonomically organized into two subfamilies: Anophelinae Grassi, 1900, and Culicinae Meigen, 1818 [3,4], with records of occurrence in most continents and bioregions, with the exception only in permanently frozen areas [5], and being particularly recognized for their great importance in transmission cycles of various infectious agents on a global scale, with this factor being the main driver of most studies and investigations carried out on this group of insects [6].

However, despite its great medical-epidemiological relevance, there are still few studies that address the evolutionary relationships between these organisms. In this context, taxonomic knowledge, as well as the survey of hypotheses about the phylogenetic relationships between mosquitoes, constitute fundamental tools in understanding basic patterns associated with aspects related to their ecology, such as specialization in larval habitat [7], vector competence [8,9], and population dynamics [10,11].

Most of the studies and investigations carried out focus on evaluating the epidemiological potential of mosquitoes, especially the genera *Aedes* Meigen, 1818, *Anopheles* Meigen, 1818, and *Culex* Linnaeus, 1758. However, it is important to consider that factors such as the increased deforestation and the unrestrained exploitation of natural resources, directly related to the consequent intensification of urbanization processes and human interference in forest ecosystems, act to significantly influence the dynamics and behavior of wild populations of mosquito species that were previously considered minor concerns, and today are increasingly gaining attention in the field of public health [12,13,14,15].

The genus *Orthopodomyia* as the only representative of the tribe Orthopodomyiini, comprises 36 species distributed according to Zavortink (1968) [16] into eight groups, largely or totally restricted, each one to a single zoogeographic region of the planet. Species of the genus can be easily distinguished from adults of other mosquito genera based on the observation of the unique ornamentation in the bands and stripes of white, silver, and gold scales in the thorax region, with some species also presenting distinctive patches of light and dark scales, particularly on the wings. Additionally, they are distinguished by the first tarsomere (T_1_) of the fore and middle legs being longer than the sum of the lengths of the four posterior tarsal segments combined (T_2–5_), with the T_4_ segment significantly shorter in relation to T_5_ [16,17,18].

Although aspects of the biology and ecological habits of representatives of the genus are not well known, it is generally observed that the immature stages are found in a variety of habitats, such as tree holes, bamboo stumps and internodes, bromeliad axils, Heliconia flower spathes, and occasionally in artificial containers [16,19]. The larval stages apparently feed by filtering microorganisms and small particles removed from the water, and the pupal stage takes five to eight days for the adult to emerge, which in turn is essentially wild and apparently presents predominantly nocturnal activity [16].

Given the widespread lack of information on the vectorial capacity of *Orthopodomyia,* it is assumed that representatives of the genus play a role in the transmission cycles of avian arboviruses due to the ornithophilic behavior exhibited by females [16,20], with only two species, *Orthopodomyia albipes* Leicester, 1904, and *Orthopodomyia andamanensis* Barraud, 1934, belonging to the oriental *Alpibes* group, which are also known to feed on humans [16]. In general, species of the genus are not recognized as vectors of medical-epidemiological importance, largely due to the difficulty of collection and the lack of records of their involvement in the transmission of infectious agents to humans and domestic animals [16]. However, the species *Orthopodomyia signifera* Coquillett, 1896, in particular, in addition to demonstrating strong ornithophilic behavior, has also been reported as a competent vector for transmission of *Eastern Equine Encephalitis virus* (EEEV) and *Western Equine Encephalitis virus* (WEEV) [21], with records of detection of these viruses from the species in regions of Mexico [22] and the United States [23].

The evolutionary relationships of *Orthopodomyia* in relation to other genera of Culicidae are not completely understood, and are mainly based on morphological aspects of the larval and adult stages [24,25]. These former studies had classified Orthopodomyiini along with Aedeomyiini, Culisetini, Ficalbiini, Toxorhynchitini, Uranotaeniini and Hodgesiini as non-related groups [3,25]. New technologies allowed for some studies to evaluate the evolutionary positioning of *Orthopodomyia* based on few molecular data, which in turn placed *Orthopodomyia* close to *Coquillettidia* Dyar, 1905 genus, and these two related to the Aedini tribe [6]. The increasing use of genomic tools applied to evolutionary studies, particularly of arthropods of medical-epidemiological importance, has significantly contributed to the development of control strategies, mainly based on evaluating properties related to the population variability of vectors and the co-evolutionary association between them and the infectious agents they can harbor and potentially transmit [26,27,28]. In this context, the use of molecular markers from genomic sets such as the mitochondrial genome, particularly applied in the development of taxonomic and evolutionary studies of metazoans, has been increasingly consolidated, due to factors such as its high rate of accumulation of mutations (particularly synonymous), absence of introns and recombinant processes, maternal uniparental inheritance, and easy amplification due to its large number of copies per cell compared to the nuclear genome (nDNA) [29,30].

The mitochondrial genome (mtDNA) consists of a compact double-stranded circular molecule, with a length ranging from 15 to 20 kb in insects, consisting of 37 functional subunits, including 13 protein-coding genes (PCGs), 22 transport RNAs (tRNAs), and 2 ribossomal RNAs (rRNAs), in addition to a final portion rich in Adenine and Thymine (A + T), associated with replicative processes [31]. Some of its main regions used in genomic investigations are active in protein coding, and belong to complexes I (*NADH* 4 and 5), III (*CytB*), and especially IV (*COI* and *COII*) [32], with some of these, specially *COI*, considering their 5’ terminal portion, being named DNA barcodes, based on the proposal that they can be used as universal identification markers of metazoans at higher levels of genus and species [33]. However, with significant advances in the development of genomic sequencing methodologies and bioinformatics tools, it is now possible for rapid and complete characterization of the mitochondrial genomes, particularly of mosquito species, supporting studies in population genetics and molecular taxonomy on the representatives for both subfamilies, Anophelinae [11,34] and Culicinae [35,36,37,38,39,40,41,42,43].

To date, studies on the application of molecular markers for identifying species and reconstructing the evolutionary relationships of *Orthopodomyia* have focused mainly on the *Signifera* (Holarctic) and *Albipes* (Oriental) groups, and as for the available mitochondrial data, existing information is limited to partial sequences, mainly from the *COI* gene [44]. Therefore, considering the great potential of the applicability of molecular methods and markers such as the mitochondrial genome in the development of taxonomic and evolutionary investigations, especially of mosquitoes, this study presents the characterization of the first complete mitochondrial genome of a Neotropical species of the genus *Orthopodomyia* found in the Brazilian Amazon region: *Or. fascipes*, using High Throughput Sequencing (HTS), and considers the reconstructing of the Culicidae phylogeny based on the maximum totality of coding regions of the investigated genome.

## 2. Materials and Methods

### 2.1. Sample Collection and Total DNA Extraction

**Figure 1:**
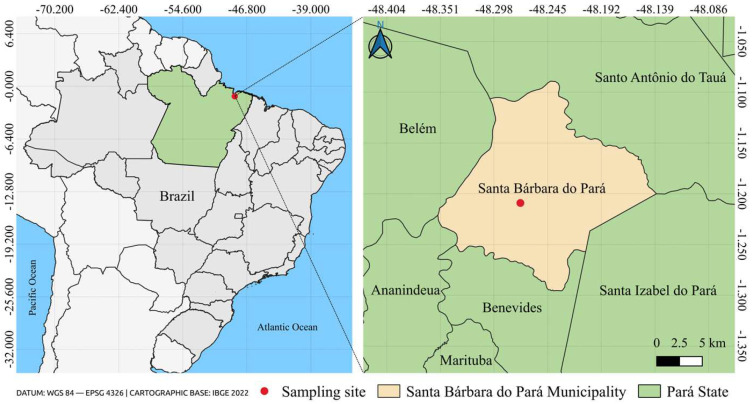
Location map of the municipality of Santa Bárbara do Pará demarcating the collection site of the investigated species. This Figure was created using the software QGIS v.3.10.4 (available at https://qgis.org/en/site/, accessed on 30 March 2024) in conjunction with the IBGE 2022 cartographic database (available at https://www.ibge.gov.br/, accessed on 30 March 2024).

The specimens of *Or. fascipes* were collected during ecoepidemiological expeditions conducted in the ecological settlement Expedito Ribeiro (1°12′32.4″ S 48°16′18.2″ W) (Figure 1), located in the municipality of Santa Bárbara do Pará, State of Pará, Brazil, from 17–19 March 2021, under authorization granted by the Biodiversity Authorization and Information System of the Ministry of the Environment (SISBIO/IBAMA), registration no. 56504-6. The collection procedures involved the installation of larvitraps made from the bark of the fruit of the chestnut tree species *Bertholletia excelsa*, locally known as *Castanha-do-Pará*, in shaded, poorly lit areas protected from rain, being left in place for five days until they were retrieved, and transported to the Medical Entomology laboratory of the Arbovirology and Hemorrhagic Fevers Section of the Evandro Chagas Institute (IEC/SVSA/MS) in the city of Ananindeua, State of Pará, Brazil (1°22′32.8″ S 48°23′03.8″ W). The immature forms collected were reared in entomological cages in the laboratory until the emergence of adults, which were subsequently identified with the assistance of the taxonomic keys developed by Lane (1953) [17] and Zavortink (1968) [16], individualized in cryogenic tubes, and stored in a freezer at −70 °C for preservation.

A single female specimen of the species *Or. fascipes* was reserved and macerated together with 360 µL of phosphate saline solution (PBS) using a 3 mm diameter stainless steel sphere in the TissueLyzer II equipment (Qiagen, Hilden, Germany). Then, 180 µL of the supernatant were reserved to carry out the total DNA extraction procedures, using the commercial kit DNeasy Blood & Tissue (Qiagen, Hilden, Germany). Subsequently, the extraction products were quantified using the dsDNA Hs Assay kit (Life Technologies, Waltham, MA, USA) on Qubit 2.0 fluorometer equipment (Life Technologies, Waltham, MA, USA), following the protocol recommendations established by the manufacturer.

### 2.2. Genomic Library Preparation and Sequencing

Total DNA extraction products were standardized to a concentration of 0.2 ng/µL and then fragmented and labeled with adapter sequences (i7 and i5) for post-sequencing identification using the Nextera XT DNA Library Preparation kit (Illumina, San Diego, CA, USA) according to the manufacturer’s recommendations. Subsequently, the genomic libraries obtained were quantified using the Qubit 2.0 fluorometer equipment (Life Technologies, Waltham, MA, USA) and qualitatively evaluated using the High Sensitivity DNA analysis kit (Agilent Technologies, Santa Clara, CA, USA) on the BioAnalyzer 2100 equipment (Agilent Technologies, Santa Clara, CA, USA). Finally, the libraries were sequenced using the NextSeq 500/550 High Output v.2.5 kit (Illumina, San Diego, CA, USA) in 300 cycles (2 × 150) on the NextSeq 500 platform System (Illumina, San Diego, CA, USA).

### 2.3. Data Processing and mtDNA Characterization

The sequencing files obtained were initially subjected to quality assessment using Fastp v.0.23.4 [45], configured to remove adapter sequences and reads with *Phred quality* base < 20 and length less than 50 nt. Subsequently, assembly was performed *de novo* using MEGAHIT v.1.2.9 [46] in its standard configuration (*k-mers* of 21, 29, 39, 59, 79, 99, 119, and 141 nt). The contig corresponding to the mtDNA of the investigated species was identified using DIAMOND v.2.1.9.163 [47] (Blastx modality, *e-value* of 10^−5^) and Krona v.2.8.1 [48], and manually inspected using Geneious v.11.1.5 [49]. The mitochondrial sequence obtained was annotated using the *MITOchondrial genome annotation Server* (MITOS) [50] and circularized by identifying overlaps between the ends using Blastn v.2.15.0 [51]. Coverage metrics by refmap and the circular structural representation of the genome were obtained using Bowtie2 v.2.5.3 [52] and CGview [53], respectively. In turn, the nucleotide composition and relative use of synonymous codons (RSCU) metrics were obtained using MEGA v.10.2.6 [54] and Geneious v.11.1.5, and the general nucleotide composition biases were estimated using the formulations AT skew = (A% − T %)/(A% + T%) and GC skew = (G% − C%)/(G% + C%) [55]. Additionally, the codon usage bias based on the effective number of codons (ENc), as well as the composition metrics GC12 (average between GC contents of the first and second codon positions), GC3 (GC content of the third codon positions), and GC3s (GC content of the third position in synonymous codons) were obtained using CodonW v.1.4.4 [56]. The evaluation of the evolutionary pressure acting on PCGs was carried out based on the calculation of the ratio between non-synonymous (*dN*) and synonymous (*dS*) substitutions (ω = *dN*/*dS*) using the CodeML application included in the PAML v.4.9j [57]. Finally, the graphs illustrating the results obtained were produced using the R v.4.3.3 [58], and the libraries *circlize* v.0.4.16 [59], *ggplot2* v.3.5.0 [60], *pheatmap* v.1.0.12 [61], and *reshape2* v.1.4.4 [62].

### 2.4. Phylogenetic Analysis

Phylogenetic analyses were performed based on the use of all 13 PCGs from the newly sequenced genome, along with another 53 mtDNAs, including assembled reference sequences and sequencing data (*rawdata*) available in the GenBank and SRA repositories (NCBI) (Table S1). Initially, the sequences were aligned using the MAFFT v.7.520 algorithm [63] and then manually inspected using Aliview v.1.28 [64]. The nucleotide substitution saturation of the set of aligned sequences was evaluated using the test developed by Xia et al. (2003) [65] in DAMBE v.7.3.11 [66]. The graphical representation of transitions and transversions in relation to genetic distance (model K80) was represented using *ape* library v.5.7.1 [67] implemented in R language. Then, using IQ-TREE v.1.6.12 [68], the best nucleotide substitution model (GTR + F + R5) was determined according to the Akaike information criterion (AIC). Subsequently, the phylogenetic signal was evaluated [69], and the phylogeny was reconstructed using the Maximum Likelihood method, with support values (bootstrapping) defined for 1000 replicates. In a parallel analysis, the phylogeny was also reconstructed using the Bayesian Inference method in MrBayes v.3.2.7a [70] in two independent and simultaneous runs, each with four chains (three hot chains and one cold one) and established for 1,000,000 generations, with sampling every 100 generations, and calculation of posterior probabilities after an initial relative burn of 25%. Convergence and estimated sample size (considering *Ess* > 200) were monitored using Tracer v.1.7.2 [71], and the obtained topologies were visualized using FigTree v.1.4.4 [72], with rooting based on the definition of the midpoint of the distances. *Dixella aestivalis* Meigen, 1818 (Diptera: Dixidae), was intentionally included in this analysis to define the anchoring group of the topologies, as it belongs to a group morphologically and phylogenetically related to Culicidae, and shares common ancestry with Chaoboridae and Corethrellidae, all belonging to the superfamily Culicoidea, suborder Culicomorpha [1,2].

## 3. Results

### 3.1. Assembly, Genomic Organization and General Composition of the Sequence Obtained

**Figure 2:**
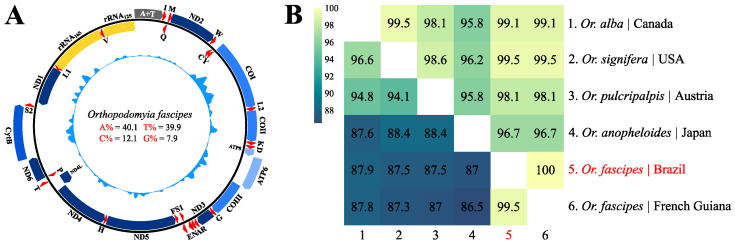
(**A**) Structural representation of *Or. fascipes* mtDNA. Internal values indicate the content of nucleotide bases. The blue inner graph indicates the distribution of genomic coverage by region. The blue, red, yellow, and gray blocks indicate the PCGs, tRNAs, rRNAs, and A + T region, respectively. Genes outside and inside the circle have forward and reverse transcription directions, respectively. (**B**) Heatmap of nucleotide/amino acid identity between taxa of the genus *Orthopodomyia*, based on the barcode region of the *COI* gene. The lower and upper triangles contain the percentages of nucleotide and amino acid identity, respectively. The taxon highlighted in red indicates the obtained sequence.

A total of 62.5 million paired reads were generated from the genomic sequencing of the investigated species, with approximately 93.1% being suitable for subsequent analysis after the quality control stage. Following genomic assembly, 623,807 contigs were obtained with lengths ranging from 200 to 1,236,672 bp (N50 = 667 bp). The contig corresponding to the mitochondrial genome of *Or. fascipes* (GenBank ID: PP749023) was 15,598 bp long, with an average coverage of 418.5× (7×~2506×), comprising 61,744 mapped reads (0.10% of the total reads generated), and was structurally organized in a compact circular molecule consisting of 37 functional subunits: 13 PCGs, 22 rRNAs, and two rRNAs, with a clear absence of inversion events and/or translocation of genes along the coding chains, including a final portion corresponding to the replication control region rich in Adenine and Thymine (A + T) (Figure 2A, Table S2). The taxonomic identification of the sequence obtained was confirmed with a comparison based on the 5’ terminal portion of the *COI* gene (639 bp) with other sequences of the genus available in the GenBank repository (NCBI) (Table S1), resulting in nucleotide and amino acid identities of 99.5% and 100%, respectively, for the investigated species (Figure 2B).

**Figure 3:**
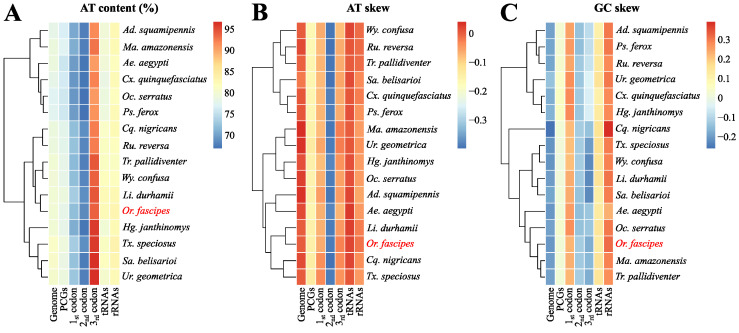
(**A**) AT% composition, (**B**) AT-skew, and (**C**) GC-skew of 16 previously characterized mosquito mtDNAs, including *Or. fascipes* (highlighted in red). Hierarchical groupings (clusters) of species (*y*-axis) are established based on the quantity of each metric per region evaluated (*x*-axis). The scales, particularly AT-skew and GC-skew, consider the real (non-normalized) values obtained in each analysis.

The sequence obtained presented nucleotide composition metrics and biases following the pattern typically observed for other species of mosquitoes with previously characterized mtDNA (Figure 3A–C, Table S3), exhibiting an AT content of 80% when evaluating the total genome (including the A + T region) and known patterns of asymmetry, with positive AT-skew and negative GC-skew, indicating the existence of higher proportions of Adenine and Cytosine in relation to Thymine and Guanine along the majority chain. Excluding the A + T region, the sequence contains 136 non-coding bp distributed in 21 intergenic regions, ranging from 1 to 21 bp (Table S2).

Most of the 22 tRNA subunits identified throughout the obtained sequence could be arranged in the typical “cloverleaf” secondary structure, with the exception of only *tRNA^Ser1^*, which, as observed in previously described mtDNAs, presented a reduction in the dihydrouridine arm (DHU), being replaced by an equivalent DHU loop (Figure S1). The total length considering the 22 concatenated tRNAs was 1487 bp, with an AT content of 80%, positive AT/GC-skews, and 19 base incompatibilities (18 GU and 01 UU). Additionally, concatenated rRNA subunits totaling 2172 bp exhibited negative and positive AT/GC-skews and structural arrangements akin to those found in other mosquito species (Figure S1).

### 3.2. Description of Protein-Coding Genes (PCGs)

Maintaining the classic pattern of genomic organization in mosquitoes, nine PCGs presented forward transcriptional sense (*ND2*, *COI*, *COII*, *ATP8*, *ATP6*, *COIII*, *ND3*, *ND6*, and *CytB*) and four reversed sense (*ND5*, *ND4*, *ND4L*, and *ND1*), varying in length (excluding stop codons) from 159 bp (ATP8) to 1734 bp (ND5), and AT content from 70.4% (*COI*) to 86.5% (*ND6*). With the exception of just *COI* (TTG), all other PCGs presented start codons in the ATN pattern, more precisely ATG (*ATP6*, *COII*, *COIII*, *ND3*, *ND5*, *ND4*, *ND4L*, and *CytB*) and ATT (*ND2*, *ATP8*, *ND6*, and *ND1*), and the complete TAA-type stop codon terminated the coding chain in 12 PCGs, except in *COII* (TAG) (Table S2).

**Figure 4:**
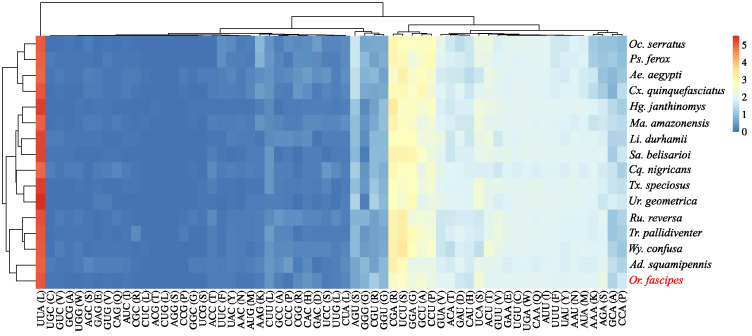
Relative use of synonymous codons of *Or. fascipes* (highlighted in red) in comparison to other representatives of the Culicinae subfamily. Hierarchical groupings (clusters) of species (*y*-axis) are established based on the RSCU of each codon (*x*-axis), with indications regarding the scale values.

Disregarding stop codons, the analysis of the relative use of synonymous codons (RSCU) recorded 3732 codons in use by the PCGs of *Or. fascipes*, expressing 58 of the 62 amino acid triplets predicted for the mitochondrial genetic code of invertebrates, with GCG (Alanine), CGC (Arginine), CUG (Leucine), and AGG (Serine) were also absent. When comparing the RSCU metrics of *Or. fascipes* with those of other species of Culicinae, it is observed that there is a pattern in the expression of amino acids, where the majority of codons whose third base is Adenine or Uracil (Thymine) are expressed at a higher frequency (mostly with RSCU > 1) than those ending in Cytosine or Guanine, taking as an example the triplets UUA (RSCUµ = 5.14) and UUG (RSCUµ = 0.19), which although expressing the same amino acid (Leucine), showed significantly different frequency metrics (Figure 4, Table S4).

**Figure 5:**
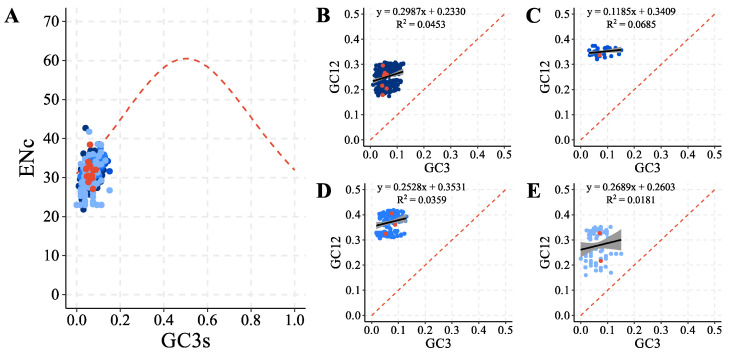
(**A**) ENc-GC3s graph. The red dashed line indicates the curve expected when codon usage bias is affected only by mutation pressure. GC12-GC3 neutrality plots are arranged in the order of mitochondrial complexes: (**B**) I (*NADH*), (**C**) III (*Cytb*), (**D**) IV (*COX*), and (**E**) V (*ATP*). In the graphs, each blue point (varying in tone according to the complex) represents a PCG independent of the Culicinae mtDNAs used as a reference in the analyses. PCGs of *Or. fascipes* are highlighted in red.

The analysis of codon usage bias based on the effective number of codons (ENc) and considering all PCGs of taxa representing the subfamily Culicinae, including the sequence recently obtained, resulted in an average of 30.4 (21.8~42.7) (Table S5), reflecting a strong trend in the use of synonymous codons (ENc ≤ 35). The evaluation of the influence of the GC content present in the third base of the synonymous codons (GC3s) on the ENc resulted in values mostly close to the base and far from the standard curve (Figure 5A), indicating that not only the mutation pressure, but also factors such as natural selection pressure may probably be involved in the formation of codon bias, particularly of the evaluated species. Additionally, the action of the main evolutionary forces that act on PCG complexes could be better observed from a neutrality analysis based on the correlation between GC12 content (average between GC content of the first and second codon positions) and GC3 (GC content third codon position). The regression analysis between these two metrics resulted in positive correlations for all PCG complexes, being weak and significant in complexes I (*NADH*, *r* = 0.2130, *p* < 0.05) and IV (*COX*, *r* = 0.1896, *p* = 0.0334) and weak and non-significant in complexes III (*CytB*, *r* = 0.2617, *p* = 0.0940) and V (*ATP*, *r* = 0.1345, *p* = 0.2223), still presenting slope coefficients lower than 0.5 (Figure 5B–E), suggesting a greater impact of natural selection pressure when compared to mutation pressure.

**Figure 6:**
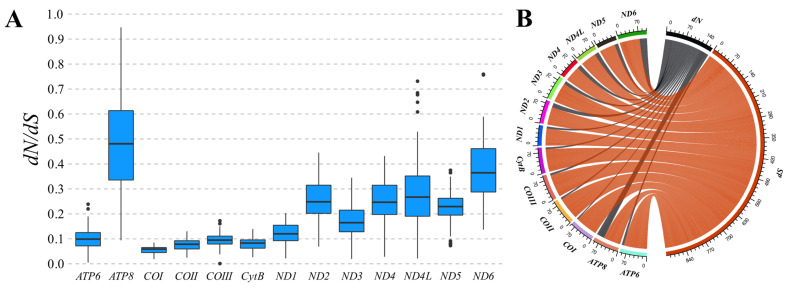
(**A**) *dN*/*dS* ratios calculated from pairwise analyses between 17 Culicinae mtDNAs. The ω ratios and PCGs are arranged along the y and x axes, respectively. (**B**) Representation of the proportion of non-synonymous (*dN*) (gray strings) and synonymous (*dS*) (red strings) substitutions that occur in each PCG. This Figure serves as a visual complement to (**A**), indicating that the accumulation of synonymous substitutions is predominantly greater in all Culicinae PCGs, directly influencing the observed negative (or purifying) selection pressure (ω < 1).

The evolutionary pressure that acts on the PCGs of the investigated species and other representatives of Culicinae could also be evaluated based on the estimate of the proportions of non-synonymous to synonymous substitutions (*dN*/*dS*). The results obtained indicate that the different coding regions are evolving globally under the influence of negative (or purifying) pressure (ω < 1), with proportions ranging from 0.0186 ± 0.0847 in *COI* to 0.0941 ± 0.9467 in *ATP8* (Figure 6A). Purifying selection was particularly strong (ω_µ_ < 0.1) on the PCGs of complexes III and IV and on the *ATP6* subunit belonging to complex V. In turn, *ATP8* and the genes belonging to complex I showed higher *dN*/*dS* ratios (0.1 < ω_µ_ < 0.5), indicating the presence of less conservative evolutionary constraints in these regions. However, it was also observed that the rates of synonymous substitutions (*dS*) on PCGs were still significantly higher compared to the occurrence of non-synonymous substitutions (*dN*) (Figure 6B).

### 3.3. Phylogenetic Analysis

**Figure 7:**
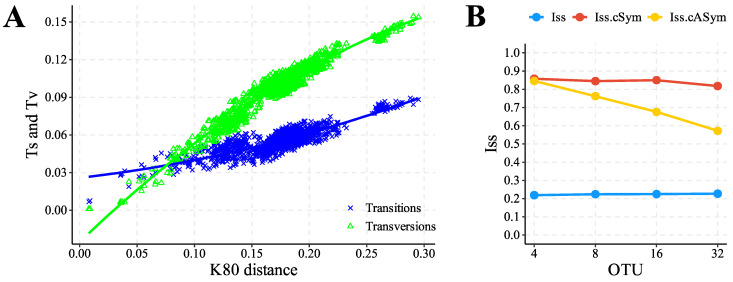
(**A**) Nucleotide substitution saturation graph of the set of evaluated sequences, considering all PCGs, demonstrating the increase and linear association of transition (*Ts*) and transversion (*Tv*) rates (*y*-axis) in relation to genetic distance (*x*-axis). (**B**) Graphical representation of the results obtained from the application of the test proposed by Xia et al. (2003) [65], statistically supported (*Iss* < *Iss.c*, *p* = 0.00001), and showing the low occurrence of nucleotide saturation in the set of sequences evaluated.

Analysis of nucleotide substitution saturation of the set of sequences evaluated resulted in the transition and transversion rates being linearly associated with genetic distance (Figure 7A). Furthermore, based on the test proposed by Xia et al. (2003) [65], performing multiple randomizations on subsets of 4 to 32 operational taxonomic units (OTUs) resulted in a saturation index (*Iss*) significantly lower (*p* = 0.00001) than the critical value (*Iss.c*), both for symmetric topologies (*Iss.cSym*) and asymmetric (*Iss.cAsym*) (Figure 7B), indicating the unsaturation of the set of sequences evaluated, and qualifying it for phylogenetic reconstruction analysis.

**Figure 8:**
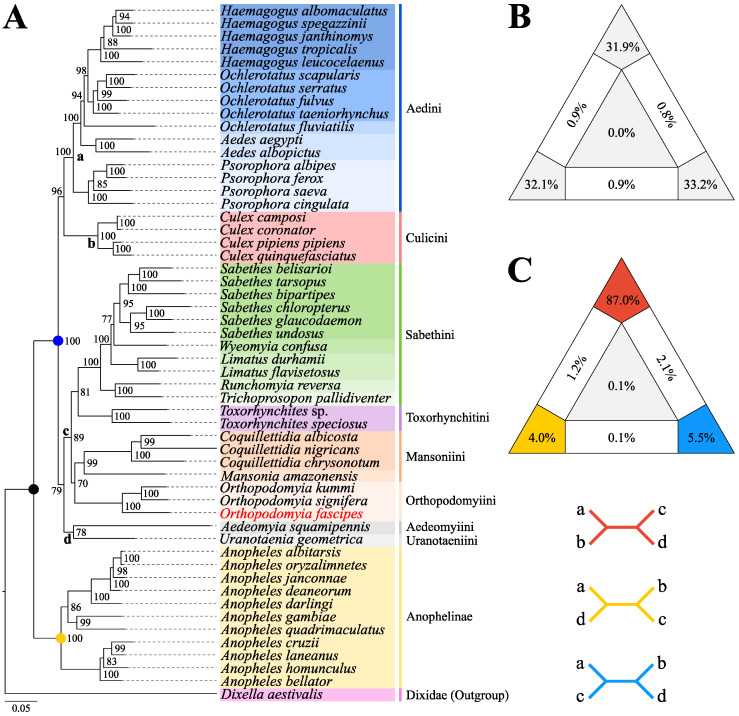
(**A**) Phylogeny reconstructed using the Maximum Likelihood method based on the 13 PCGs of *Or. fascipes* (highlighted in red) and other taxa available in the GenBank repository (NCBI). Bootstrapping support values (BS) are shown on each node. The colored dots indicate the main reconstructed taxonomic groupings: family Culicidae (black) and subfamilies Culicinae (blue), and Anophelinae (yellow). (**B**) Maximum Likelihood mapping diagram demonstrating the quality of the phylogenetic signal resulting from the quartet analysis, with 98.2% resolved quartets. (**C**) Four-cluster likelihood mapping (FcLM) diagram representing four taxon groupings in Culicinae. The analyzed groups are indicated in the topology by the letters “a” (Aedini), “b” (Culicini), “c” (Orthopodomyiini + Mansoniini + Toxorhynchitini + Sabethini), and “d” (Aedeomyiini + Uranotaeniini). The bidirectional arrows indicate different hypotheses regarding the relationships among the four clusters, with their respective colors in the diagram corresponding to the support percentages.

Reconstruction of the phylogeny using Maximum Likelihood (Figure 8A) and Bayesian Inference (Figure S2) resulted in identical topologies with high internal anchoring values, consisting of a main group, externally anchored by the taxon *D. aestivalis* and containing 53 taxa distributed in two well-structured and monophyletic clades corresponding to the subfamilies Culicinae (with 42 taxa divided into eight tribes) (BS = 100%, BPP = 100%) and Anophelinae (with 11 taxa) (BS = 100%, BPP = 100%). The evaluation of the quality of the phylogenetic signal of the set of sequences used, particularly in Maximum Likelihood phylogeny, resulted in 97.2% of resolved quartets with an approximate distribution at the vertices of the diagram (Figure 8B), demonstrating the reconstruction of well-resolved topologies and high reliability during analysis.

In both analyses, the Aedini + Culicini group was externally anchored by a subclade made up of Sabethini + Toxorhynchitini, with Mansoniini + Orthopodomyiini as its sister group, and was externally anchored by Aedeomyiini + Uranotaeniini. This phylogenetic conformation in Culicinae was strongly supported by FcLM analysis, showing a support value of 87% (Figure 8C). *Or. fascipes*, together with other taxa of its genus, constituted a reasonably well-supported subclade (BS = 70%, BPP = 96%) with representatives of the genus *Coquillettidia* and *Mansonia* Blanchard, 1901, which was corroborated by the smaller nucleotide distance of these taxa in relation to the tribe Mansoniini when compared with other groups such as Aedini, Culicini, and Sabethini (Figure S3, Table S6).

## 4. Discussion

The present study describes, for the first time, the complete mitochondrial sequence of the species *Or. fascipes*, collected during ecoepidemiological expeditions in the northeast region of the State of Pará, Brazil, and characterized using HTS. When compared with mitochondrial genomes from previously described mosquito species, the sequence obtained exhibited conserved characteristics in several aspects, including the arrangement of its 37 functional subunits in two transcription chains, the length and metrics of nucleotide composition, the arrangement of secondary structures of tRNAs, and the use of codons by PCGs [37,38,39,40,41,42,43], with no translocation events observed, especially between subunits of tRNAs [32], as evidenced in mtDNAs from representatives of the Sabethini tribe [36].

The predominance of unevenly distributed nucleotide bases, commonly resulting in a high A + T content, is a phenomenon frequently observed in insect mitochondrial genomes, significantly impacting the constitution of protein-coding regions [35,73]. In this context, in addition to the majority of *Or. fascipes* PCGs having start codons in the ATN pattern (except *COI*, which presented TTG) and complete stop codons of the TAA type (except *COII*, which presented TAG), we also observed a substantial increase in AT content in the third position of amino acid codons. These characteristics corroborate the pattern observed in previous studies characterizing the mtDNA of mosquito species [34,35,36,37,38,39,40,41,42,43], and are justified, respectively, by the occurrence of post-transcriptional polyadenylation in the formation of complete stop codons [74] and by the high availability of free ATP, which together with other NTPs, promotes an increase in the use of Adenines, especially in the third codon position, contributing positively to the efficiency of transcription processes [75].

Considering the clear pattern in amino acid expression in relative codon usage (RSCU) analysis among representatives of Culicinae, including *Or. fascipes*, it is suggested that the high AT content in the third codon position may be associated with bias in usage of synonymous codons, thus influencing the reduction in the intensity of purifying selection against deleterious mutations in this same region [35,76]. Furthermore, the effective number of codons (ENc) and neutrality analyses, used, respectively, to detect heterogeneity in the use of codons [77] and determine the main evolutionary forces [78] acting on the PCG complexes of Culicinae, showed a strong tendency in the use of synonymous codons (ENc ≤ 35), further indicating that factors such as mutational pressure and natural selection may be influencing the formation of the codon bias observed in Culicinae, with natural selection appearing to have a greater impact, as indicated by the slope coefficients obtained. Given these findings, this study complements the efforts of a previous evaluation carried out by Hao et al. (2017) [35], who obtained similar results when analyzing 50 mtDNAs from mosquitoes, although these were representatives of the Anophelinae.

Additionally, it became evident that PCGs from *Or. fascipes* and other representatives of Culicinae are evolving globally under negative (or purifying) selection pressure (*dN*/*dS* < 1), resulting in high rates of synonymous substitutions within the evaluated regions [79]. As previously noted in other studies, this purifying selection pressure was particularly stronger in complexes III (*CytB*) and IV (*COX*), while there were fewer conservative constraints and a tendency towards positive (or adaptive) selection pressure, especially in complex I (*NADH*) and *ATP8*. However, it is important to note that different rates of mutation accumulation occur among PCG complexes, depending on the role each plays in metabolic processes and in the reactions of the oxidative phosphorylation chain of cellular respiration [80].

In this study, the joint use of PCGs from 54 mitochondrial genomes, including the newly sequenced species, and two phylogenetic reconstruction approaches (ML and BI) resulted in topologies with high internal anchoring values and significant support for the basal dichotomy between the subfamilies Culicinae and Anophelinae, and also corroborated the monophyly, in Culicinae, of the tribes Aedini [40,43], Culicini [41,81], Sabethini [36,42], and Mansoniini [38,82]. However, our findings suggest that the Aedini and Culicini tribes make up a sister group of the monophyletic group formed by Aedeomyiini + Uranotaeniini + (Mansoniini + Orthopodomyiini + (Sabethini + Toxorhynchitini)), corroborating other studies that used the same genomic set in previous investigations, with the continued absence of representatives of the Orthopodomyiini tribe [37,38,39,40,83], and partially disagreeing with the results obtained in other investigations based on the use of mitochondrial markers [41].

The monophyletic grouping Sabethini + Toxorhynchitini, externally anchored by Mansoniini, is consistent with results obtained in recent studies that used mitochondrial and nuclear markers, corroborating the evolutionary positioning of these tribes [39,41,82]. On the other hand, these results contrast with previous analyses based on the observation of morphological characters and nuclear markers in a smaller sample, where Mansoniini was classified as a sister group to Aedini, as well as Sabethini in relation to Culicini, and Toxorhynchitini appeared as a basal taxon [6,25].

Furthermore, outside the clade constituted by the set of tribes initially mentioned, Aedeomyiini and Uranotaeniini were recovered in a single group, diverging in evolutionary positioning in relation to previous studies. Reidenbach et al. (2009) [6], by employing six nuclear markers to reconstruct the phylogeny of Culicidae, demonstrated that Aedeomyiini and Toxorhynchitini, externally anchored by a representative of the genus *Mimomyia* Theobald, 1903 (tribe Ficalbiini), formed a monophyletic group with Sabethini, while Uranotaeniini grouped with Culicini. These observations were later corroborated by Lorenz et al. (2021) [39] in a temporal analysis covering 102 taxa and considering the use of all 13 mitochondrial PCGs.

In contrast, the evolutionary positioning of Uranotaeniini varied according to the configuration of the mitochondrial dataset and the phylogenetic reconstruction methods used by Chen et al. (2023) [41]. In this study, the phylogeny reconstructed with the Maximum Likelihood method recovered the monophyletic grouping Uranotaeniini + Sabethini, while in the topologies obtained with Bayesian Inference, Uranotaeniini was positioned outside the group formed by Mansoniini + (Sabethini + Toxorhynchitini). Additionally, in previous studies based on mitochondrial [38,83], nuclear [82], and morphological data [25], Aedeomyiini and Uranotaeniini were consistently observed as external and basal groups in relation to the monophyletic grouping formed by all other tribes of Culicinae. In this context, although our results contribute to clarifying the evolutionary history of these tribes, we recognize the need to carry out more comprehensive investigations and broader sampling efforts, aiming to establish a solid basis for their molecular taxonomy.

In the present study, in the context of the evolutionary order, particularly of the second subclade of tribes in Culicinae, the taxon *Or. fascipes*, together with other taxa of its genus, constituted a monophyletic and reasonably well-supported grouping (BS = 70%, BPP = 96%), together with representatives of the Mansoniini tribe, which was also evidenced when evaluating the nucleotide distance metrics. The results obtained corroborate molecular studies carried out previously, including representatives of the genus, where their proximity to Mansoniini was also observed, albeit with analyses based on the use of nuclear markers and significantly different sampling of genes [6,82]. On the other hand, studies that evaluated the evolutionary positioning of Orthopodomyiini based on the observation of morphological characters considered the possibility that representatives of the tribe occupied basal positions in relation to the other tribes of Culicinae [25]. This was even supported in a molecular study carried out by Munstermann et al. (1985) [84], who, after evaluating the structure of the well-resolved polytene chromosomes of *Orthopodomyia pulcripalpis* Rondani, 1872, belonging to the *Signifera* group, suggested that the species may have gone through potentially greater evolutionary periods compared to other species of the subfamily. These observations are further supported by the fact that *Orthopodomyia* is a monotypic taxon that does not present subgeneric classifications to date, and is considered somewhat primitive since its current and wide geographic distribution may be the result of its dissemination before geological events associated with the division of Africa and South America in the Cretaceous [6].

*Or. fascipes* has three taxonomic synonyms: *longipalpis*, *townsendi*, and *bacigalupoi*, and curiously, due to the presence of characteristics similar to representatives of the Mansoniini tribe, in its first description records it was named *Mansonia fascipes* by Coquillett (1905) [85]. The species has a geographic distribution that ranges from Nicaragua to the south of Bolivia, and extends through the Brazilian Amazon region to the states of Goiás and Minas Gerais, being classified according to Zavortink (1968) [16] as one of the two representative species of the group (or section) Neotropical *Thomasina* responsible for most of the known characteristics of the group, mainly in relation to the morphological aspects of the immature stages. In this context, little is known about the initial developmental stages of *Orthopodomyia sampaioi* da Costa Lima, 1935, with the distinction between the two species being precisely evidenced only based on the morphology of the adults, and although they are basically allopatric, their distribution areas apparently overlap in the Brazilian state of Goiás.

The evolutionary affinity relationships of *Orthopodomyia* with other genera of Culicidae are largely misunderstood, and to date have been concluded to be mainly based on the observation of morphological characters [16,24,25], relying on very few studies available to assess its evolutionary position using molecular markers. In this context, the unavailability of genetic information about this group of mosquitoes is possibly due to the lack of bioecological records and their involvement in the transmission and/or maintenance of infectious agents, particularly in wild environments, neglecting their medical-epidemiological and evolutionary importance.

Taxonomy based on morphological aspects continues to be, without a doubt, the main means of classifying and validating species identification processes. However, this method is not free from possible flaws, especially when dealing with a group of organisms as diverse and ancient as the Culicidae family. The advent of molecular tools and computational analysis methods has made it possible to carry out increasingly deeper investigations into the taxonomic relationships between these organisms. In this context, the characterization and application of mitochondrial genomes have contributed greatly to the supply of information at various levels of the system. Despite this, it is evident that mtDNA may not be sufficient to recover deeper phylogenetic relationships. Therefore, integration with other markers may be appropriate, considering that this also represents a future objective in the development of evolutionary studies, especially of insects [73,86].

This study represents the first record of the complete mitochondrial genome recovery of a Neotropical species belonging to the genus *Orthopodomyia* collected in the Brazilian Amazon region. Until this study, there were no records of complete mitochondrial sequences of the genus deposited in public repositories, except for only two records of genomic sequencing data (*rawdata*) of species from the *Signifera* group obtained by Soghigian et al. [82] that are available in the SRA database (NCBI). These data were included in the analysis of this study, and although they did not constitute complete mitochondrial sequences after assembly using the *de novo* method and/or reference mapping, it was possible to recover a large part of the PCGs content.

We consider that with the increasing number of molecular taxonomy studies of Culicidae, based on the acquisition of new mtDNAs, the phylogenetic relationships observed in this study could potentially change. Consequently, it will always be necessary to extensively expand taxon sampling to reconstruct more reliable Culicidae phylogenies, focusing particularly on areas rich in high biodiversity, such as the Amazon region.

## Data Availability

All the data obtained during this study are available in the tables and figures included in the text, and in the Supplementary Materials. The mitochondrial sequence of *Or. fascipes* obtained here was deposited in the GenBank database under accession code PP749023, and the raw sequence reads generated are available in the NCBI Sequence Read Archive (SRA) database under BioProject PRJNA1116825, BioSample SAMN41551723, and SRA acession SRR29190358.

## Supplementary Materials

The following supporting information can be downloaded at: https://www.mdpi.com/article/10.3390/genes15070874/s1, Figure S1—Secondary structure of tRNAs and rRNAs of *Or. fascipes*; Figure S2—Phylogeny reconstructed by the Bayesian Inference method based on the 13 PCGs of *Or. fascipes* and other taxa available in the GenBank repository (NCBI); Figure S3—Intra/intergroup distances between four tribes of Culicinae and *Or. fascipes*; Table S1—Taxa used in the evolutionary analyzes; Table S2—*Or. fascipes* mtDNA nucleotide composition metrics; Table S3—Nucleotide composition metrics of *Or. fascipes* in comparison with other species of the Culicinae; Table S4—Relative use of synonymous codons (RSCU) of *Or. fascipes* in comparison with other species of the Culicinae; Table S5—ENc, GC12 and GC3s metrics of *Or. fascipes* in comparison with other species of the Culicinae; Table S6—Maximum Likelihood Composition nucleotide distance matrix.
