# Supplementary material for: Sequencing and Description of the Mitochondrial Genome of Orthopodomyia fascipes (Diptera: Culicidae)
Source: Genes (Basel). 2024 Jul 3;15(7):874. doi: 10.3390/genes15070874 (PMC11276460; doi:10.3390/genes15070874)
Supplement: Supplementary file 1 [file genes-15-00874-s001.zip › figure_s2.pdf]

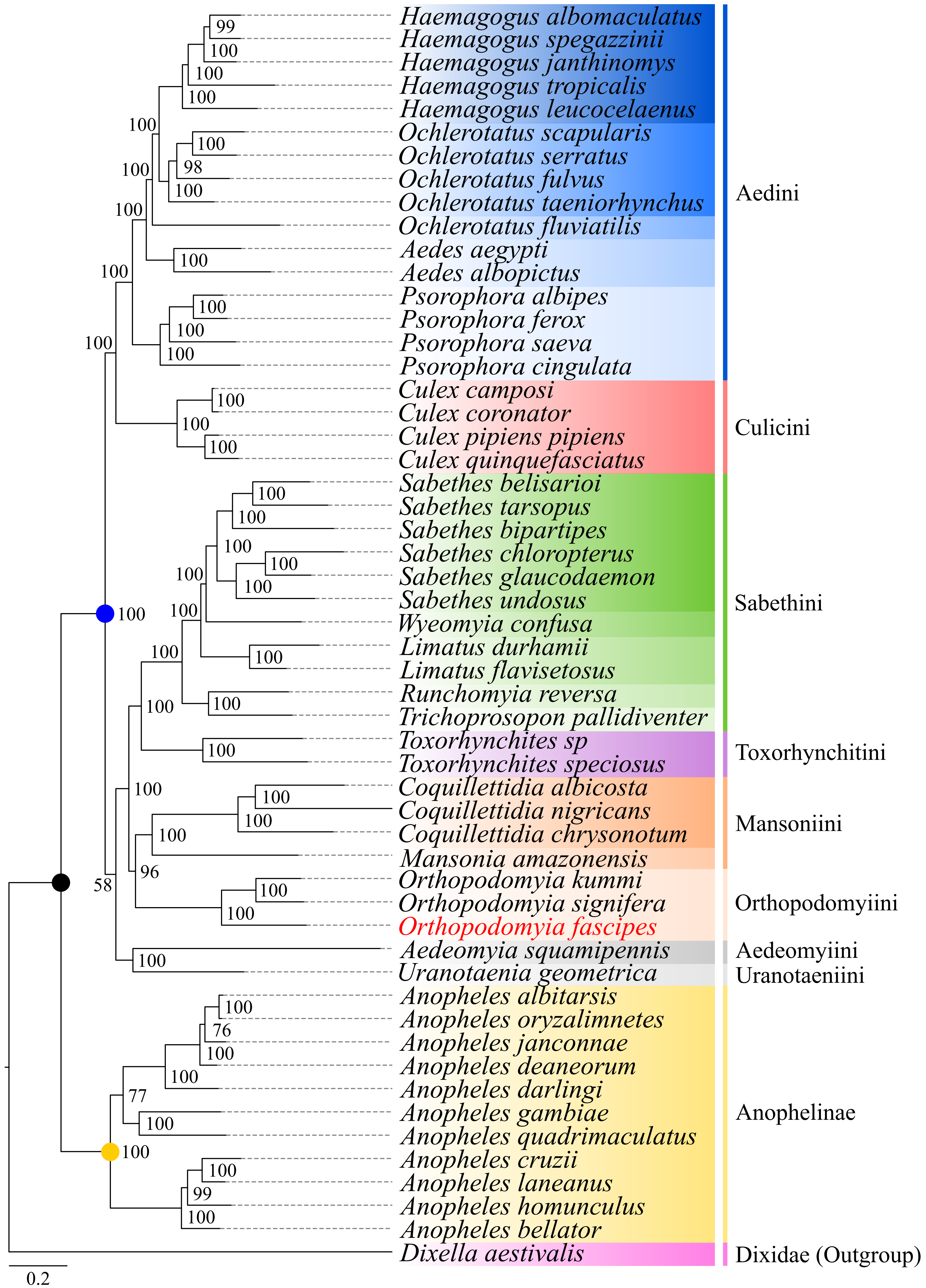

Figure S2. Phylogeny reconstructed by the Bayesian Inference method based on the 13 PCGs of *Or. fascipes* (highlighted in red) and other taxa available in the GenBank repository (NCBI). Bayesian posterior probabilities (BPP) are shown at each node. The colored dots indicate the main reconstructed taxonomic groupings: the family Culicidae (black) and the subfamilies Culicinae (blue) and Anophelinae (yellow).
