# Supplementary figures and images for: Sequencing and Description of the Mitochondrial Genome of Orthopodomyia fascipes (Diptera: Culicidae)
Source: Genes (Basel). 2024 Jul 3;15(7):874. doi: 10.3390/genes15070874 (PMC11276460; doi:10.3390/genes15070874)

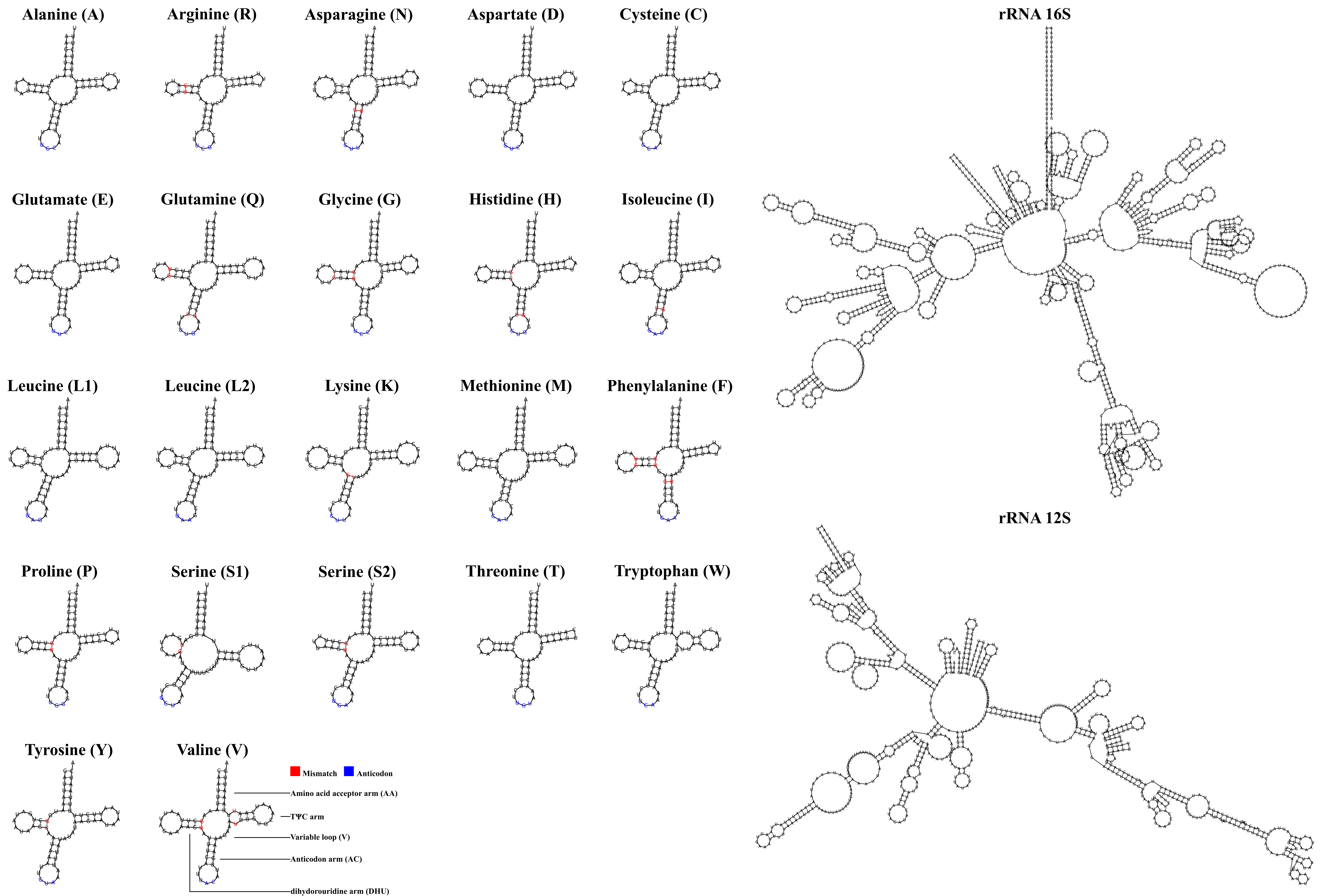

**Figure S1.** Secondary sctructures of tRNAs and rRNAs of *Or. fascipes*.

Supplement: Supplementary file 1 [file genes-15-00874-s001.zip › figure_s1.pdf]

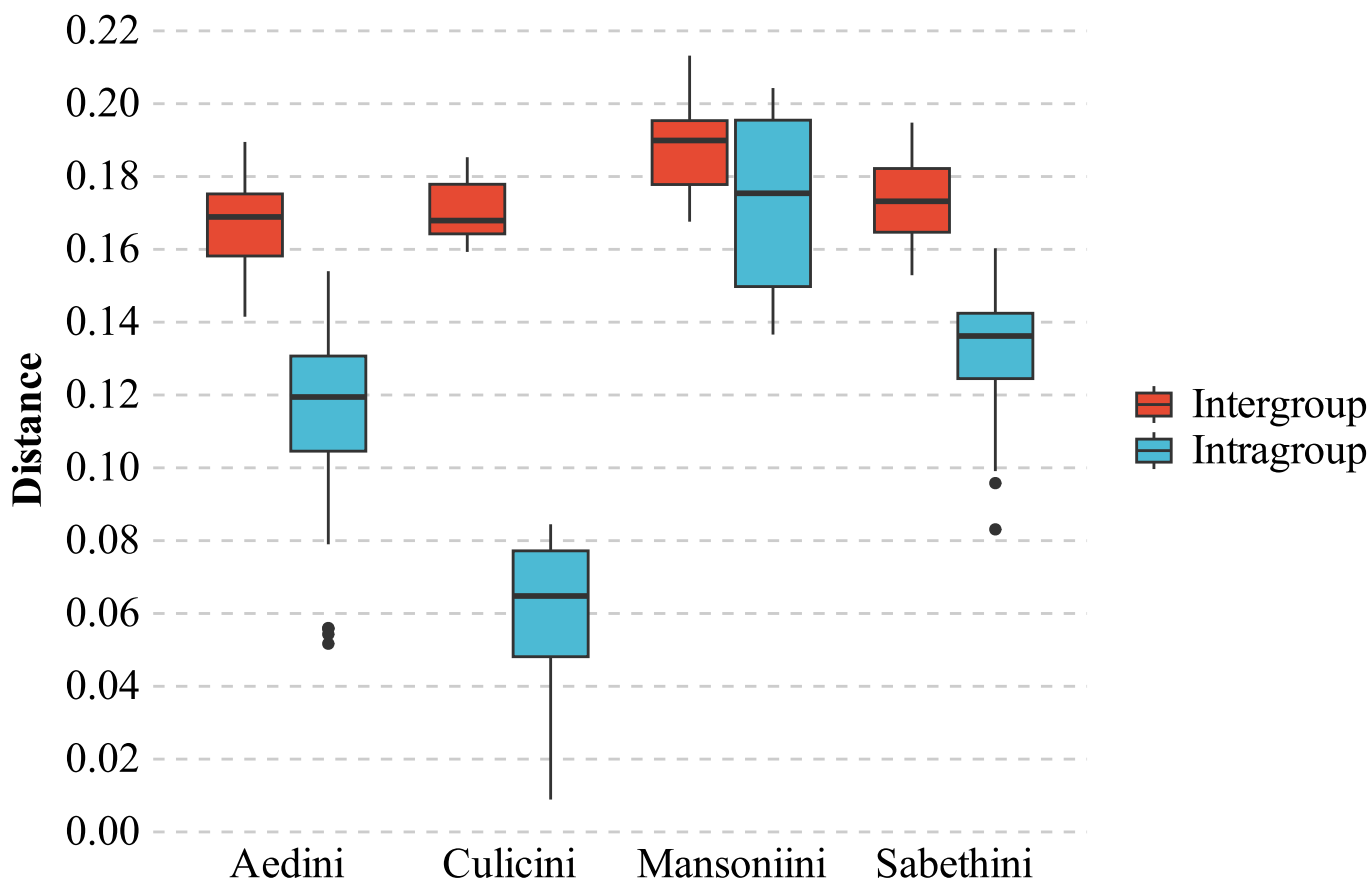

**Figure S3.** Intra/intergroup distances between four tribes of Culicinae and *Or. fascipes*.

Supplement: Supplementary file 1 [file genes-15-00874-s001.zip › figure_s3.pdf]
